# Mass drug administration trials of azithromycin: an analysis to inform future research and guidelines

**DOI:** 10.1186/s40249-025-01322-8

**Published:** 2025-07-21

**Authors:** Alex C. Kong, Anthony D. So

**Affiliations:** 1 Department of International Health, Johns Hopkins Bloomberg School of Public Health, Baltimore, MD USA; 2 Innovation + Design Enabling Access (IDEA) Initiative, Johns Hopkins Bloomberg School of Public Health, Baltimore, MD USA

**Keywords:** Mass drug administration, Antimicrobial resistance, Azithromycin, Clinical trial design

## Abstract

**Background:**

In 2020, the World Health Organization published a guideline on the use of mass drug administration (MDA) of the broad-spectrum antibiotic azithromycin to reduce childhood mortality. As MDA-azithromycin to reduce mortality is considered for expansion to more settings and populations, care must be taken to maximize benefits and reduce risks (e.g., antimicrobial resistance or AMR) of this intervention. Completed and ongoing MDA-azithromycin cluster-randomized clinical trials can provide evidence on the extent to which these benefits and risks accrue and identify practices to monitor these effects and address evidence gaps in future trials.

**Methods:**

We examined azithromycin clinical trials registered on ClinicalTrials.gov and the WHO International Clinical Trials Registry Platform from registry inception to December 31, 2023. We included trials for which azithromycin was administered for the prevention or treatment of a disease or condition that was not explicitly diagnosed or necessary for participant inclusion, and for which treatment was randomized by geographic units. We identified evidence, knowledge gaps, and trends and highlights across five domains: (1) targeting of MDA-azithromycin, (2) clinical endpoints, (3) co- and competing interventions, (4) spillover effects, and (5) AMR monitoring.

**Results:**

Of 1589 screened studies, 30 met all inclusion criteria. These trials were conducted in 13 countries, predominantly (26/30) in sub-Saharan Africa. Nearly a third (9/30) of the trials included mortality endpoints, but few (2/9) included cause-specific mortality endpoints. New evidence suggests the benefits of widening the target age group and the persistence of mortality benefits in settings with competing interventions. Published practices to ensure geographic separation of communities in different treatment arms to reduce spillover effects were not customary. We found information on AMR monitoring practices for just over half the trials (16/30). Of these, half (8/16) included both phenotypic and genotypic AMR testing, and more than half collected specimens to assess the nasopharyngeal and gut microbiomes (9/16) and tested for non-macrolide resistance (11/16).

**Conclusions:**

Further long-term MDA-azithromycin studies to determine which additional countries could benefit, interventions to accompany or replace this intervention, and the extent to which AMR spillover occurs may prove valuable as guidelines are revised.

**Graphical Abstract:**

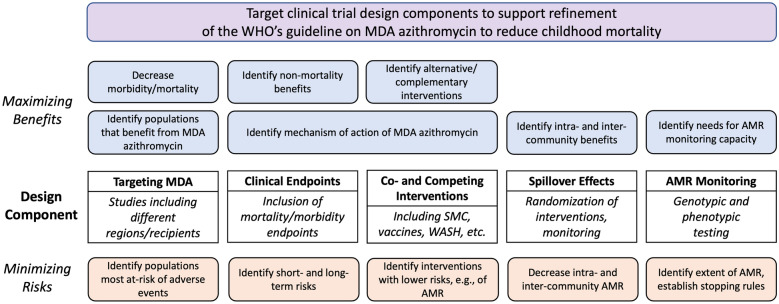

**Supplementary Information:**

The online version contains supplementary material available at 10.1186/s40249-025-01322-8.

## Background

Mass drug administration (MDA) programs in which the broad-spectrum antibiotic azithromycin is administered to an entire population or segment of a population, regardless of infection status, are critical components of strategies for the control of diseases like trachoma [1] and yaws [2]. The World Health Organization (WHO) classifies azithromycin as a Watch antibiotic under the AWaRe (Access, Watch, Reserve) framework, denoting its value as a first or second choice antibiotic for specific infections and propensity for selecting for antimicrobial resistance (AMR) [3].

In 2020, the WHO published a guideline on the use of MDA-azithromycin to promote child survival [4] based upon three key randomized clinical trials (RCTs): the Trachoma Amelioration in Northern Amhara (TANA) trial, for which childhood mortality was a secondary endpoint [5]; the Macrolides Oraux pour Réduire les Décès avec un Oeil sur la Résistance (MORDOR) trial [6]; and the Seasonal Malaria Chemoprevention Plus Azithromycin in African Children (SMCAZ) trial [7]. These RCTs produced mixed results, raising questions regarding which populations would benefit from MDA-azithromycin. However, substantive differences in methodological design precluded pooling of the three trials’ results.

Both the TANA trial in Ethiopia and MORDOR trial in Malawi, Niger, and Tanzania found that MDA-azithromycin led to statistically significant childhood mortality reductions compared to communities that did not receive azithromycin [5, 6]. The MORDOR trial observed that biannual MDA-azithromycin led to an overall reduction in mortality for children 1–59 months of age across its three sites, though much of the effect was attributable to reductions in Niger only [6]. Finally, the SMCAZ trial in Burkina Faso and Mali found no difference in its composite outcome of death or hospital admission when azithromycin was administered with seasonal malaria chemoprevention (SMC) versus SMC and placebo, though this trial was randomized at the household- rather than community-level and was powered to detect a much larger difference in its primary endpoint than the MORDOR trial (25%, versus 10% in the MORDOR trial) [6, 7].

Based on the observed benefits and risks reported in these trials and other literature, the WHO made two recommendations on MDA-azithromycin to reduce childhood mortality, both with the caveat that the available evidence was low-quality:

1. A strong recommendation against the universal use of MDA-azithromycin to reduce childhood mortality.
2. A conditional recommendation to consider MDA-azithromycin only in children between 1 and 11 months of age in sub-Saharan African settings where:
  - Infant mortality is > 60 per 1000 live births or under-five mortality is > 80 per 1000 live births *and*
  - Infant and under-five mortality rates, adverse effects related to azithromycin, and antibiotic resistance are continuously monitored, *and*
  - Existing child survival interventions could be simultaneously strengthened [4].

These recommendations outline key areas where differing degrees of uncertainty remain. The three trials used for guideline development were all conducted in countries in sub-Saharan Africa, and a subgroup analysis from the MORDOR trial suggested that the intervention conferred the greatest mortality benefits upon younger children aged 1–5 months when assessing crude effect estimates, but this analysis was not powered to detect statistically significant differences in mortality by age groups [6]. Furthermore, none of the trials considered in the drafting of this guideline had targeted treatment to 1–11-month-old children only, thus the effectiveness of targeting just this age group was unknown. Although there were key differences in the design of the SMCAZ trial compared to the TANA and MORDOR trials, its negative results suggest the importance of considering the effects of co-interventions (i.e., interventions administered with azithromycin) and competing interventions (i.e., interventions that are not administered with azithromycin, but that might be present outside a trial) in amplifying or nullifying MDA-azithromycin’s mortality-reducing benefits. This could help to determine MDA-azithromycin’s mechanism of action for reducing childhood mortality and to determine where interventions could work in synergy or supplant azithromycin.

The possibility of MDA-azithromycin leading to or exacerbating AMR holds important implications for both direct and indirect beneficiaries of this intervention and the sustainable effectiveness of this approach. Some early studies found that annual MDA-azithromycin campaigns for trachoma can cause transiently elevated AMR, though this decreased after cessation [8, 9]. Additionally, the impact of AMR caused by MDA-azithromycin can be difficult to discern due to “spillover” effects, whereby the benefits (e.g., mortality reduction and reduced infections) and the risks (e.g., AMR) can spread from a treated population to an untreated one if sufficient precautions are not taken to adequately separate these populations under study [10]. The benefits and risks of MDA-azithromycin, both in participating and neighboring communities, should be considered in clinical trials and the programs they inform, whether for trachoma, mortality, or other indications [11].

Since the WHO published this guideline, several community-randomized trials using MDA-azithromycin have been designed and initiated. In this review, we analyzed both completed and ongoing randomized community trials using MDA-azithromycin to identify which evidence gaps have been filled or persist since the original WHO guideline’s publication and to identify practices—from trials with mortality and non-mortality endpoints—that could inform the development of future MDA-azithromycin trials and support evidence generation. As the WHO seeks to review its guidelines in the coming years, finding the clinical equipoise between benefits and risks can ultimately impact wider implementation recommendations and ensure that decisions to use MDA-azithromycin to reduce childhood mortality adequately account for these potential trade-offs, both short- and long-term, for individuals and communities.

## Methods

### Search strategy and inclusion and exclusion criteria

We conducted a search using the term “azithromycin” to identify all clinical trials that included azithromycin as an intervention, that were registered in ClinicalTrials.gov and the WHO International Clinical Trials Registry Platform (ICTRP) between inception and December 31, 2023, and that had not been withdrawn or terminated. We only included trials in which azithromycin was administered to treat or prevent non-diagnosed diseases or conditions (see Table 1). By including clinical trials that did not track mortality as an endpoint, the understanding of other key endpoints like AMR surveillance could be better captured. Only clinical trials randomized at the community level were included in this review, as the community-level benefits of the intervention cannot be fully ascertained when randomized below this geographic unit of analysis [12]. We excluded trials in which azithromycin was administered prophylactically to minimize the risk of infections from procedures such as routine surgeries and Cesarean sections. The indication for such uses was at the individual, not community level.

**Table 1:** Inclusion and exclusion criteria for azithromycin clinical trials

Clinical trial element	Inclusion criteria	Exclusion criteria
Intention to treat based on clinical diagnosis	Azithromycin is administered for a disease or condition which has not been explicitly diagnosed across the study population AND Administered in a population for which having a certain disease or health condition is not a requirement for inclusion	Azithromycin is exclusively administered for diagnosed infectious diseases OR Administered in a population for which having a certain disease or condition is a requirement for inclusion OR Administered for surgical prophylaxis
Randomization of intervention by geographic area	Unit of randomization is at the geographic level (e.g., community, village, catchment area, etc.)	Unit of randomization is at the individual or household level OR Intervention is not randomized (e.g., cohort studies)

We also reviewed published resources related to the included trials, including clinical trial protocols and peer-reviewed journal articles linked to the clinical trial registry entries. We supplemented this with a search of the included clinical trials’ identification codes on PubMed and reviews of published journal articles’ bibliographies to ensure the review of relevant journal articles that were not listed in the registries.

### Quality assessment of included literature

To ensure the inclusion of high-quality literature, we employed a targeted selection approach focused on reputable and well-regarded sources. These sources included peer-reviewed literature, published protocols, and grey literature. Clinical trial information and supporting documentation, such as trial protocols, were drawn from established trial registries. Our analysis of peer-reviewed journal articles included scrutiny of methodologies and findings to include relevant and reliable evidence. Additional guidelines and datasets used in this analysis were sourced from internationally recognized organizations including the WHO and other United Nations organizations. Where possible, data from multiple sources were used to triangulate information and enhance confidence in the findings.

### Evidence extraction and analysis

**Fig. 1:**
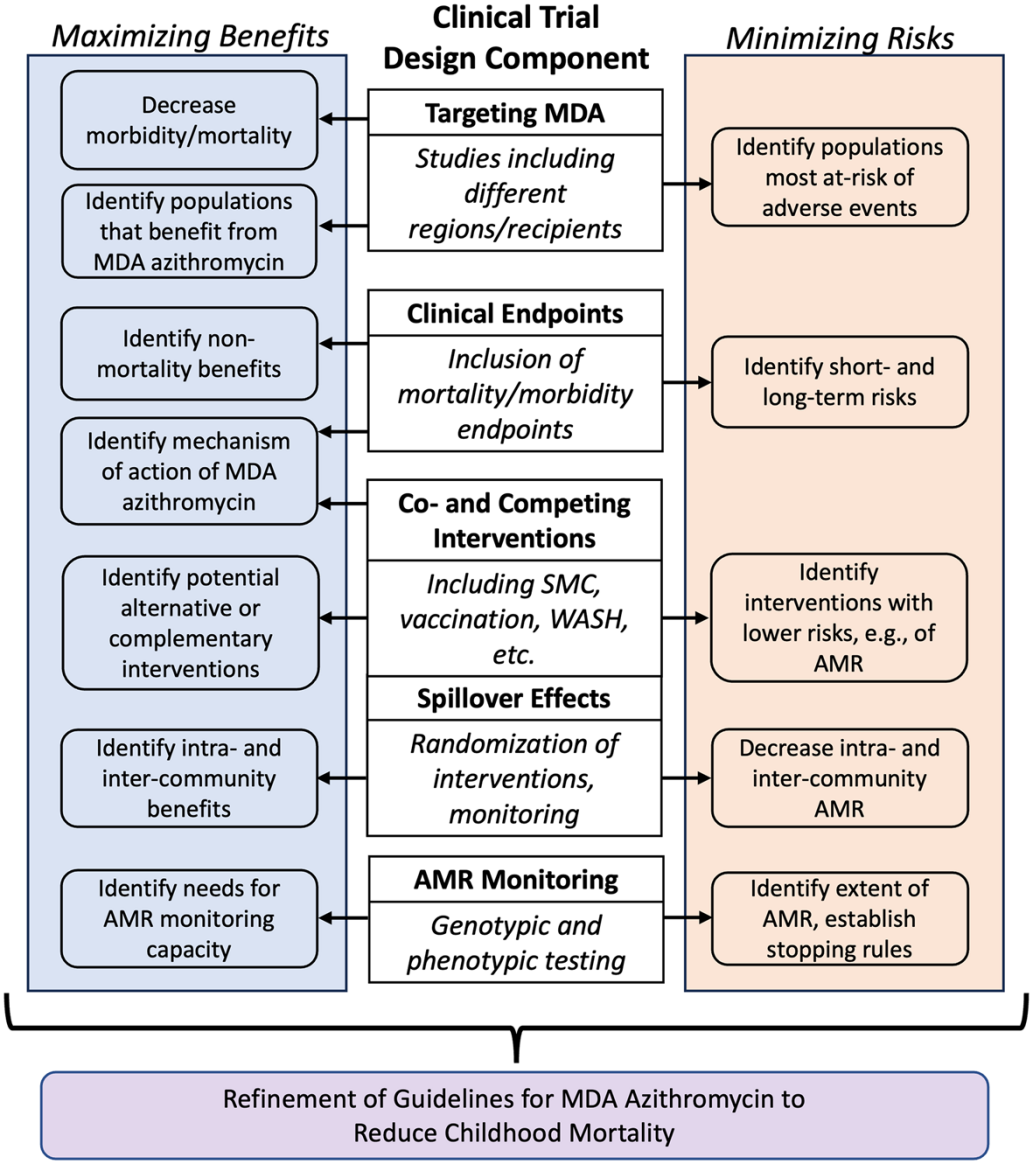
Five key components for consideration in designing clinical trials involving the mass administration of azithromycin. The boxes on the left indicate ways in which these design components can help to maximize the benefits of MDA-azithromycin or other interventions, and the boxes on the right indicate ways in which the components can help to minimize the risks of MDA-azithromycin. *AMR* Antimicrobial resistance, *MDA* Mass drug administration, *SMC* Seasonal malaria chemoprevention, *WASH* Water, sanitation, and hygiene.

During this review, we established five key dimensions where researchers could potentially design studies to collect data that better maximize the benefits and minimize the risks of MDA-azithromycin and for which we assessed evidence relevant to the WHO guideline. These dimensions included the targeting of MDA-azithromycin, consideration of co-interventions and competing interventions, the inclusion of mortality and morbidity clinical endpoints, monitoring and control of spillover effects, and monitoring for AMR (Fig. 1).

The rationale for including each dimension and types of data extracted for each is described below.

#### Targeting MDA

Community trials using MDA-azithromycin are essential for generating evidence to identify the populations that may benefit from this intervention. We collected information on where trials were conducted, the number of participants, and the age range of participants to discern which populations were included and excluded. To provide context on the background mortality rates in the countries where trials were conducted, we used baseline data from the United Nations Inter-agency Group for Child Mortality Estimation (UN IGME) 2023 Report for the years in which initial trial enrollment began [13]. We also identified other countries with high mortality rates using point estimates and upper bounds—the use of the latter accounting for variation in mortality rates at a subnational level—to determine which additional countries might benefit from MDA-azithromycin.

#### Clinical endpoints

The selection of primary and secondary endpoints is used to establish research priorities and to determine the sample sizes necessary to detect differences between intervention groups. Different endpoints provide different insights: some endpoints can help to discern how MDA-azithromycin reduces mortality while others can identify risks of MDA or characterize the feasibility of implementing this intervention. We collected information on the self-reported primary and secondary endpoints for each included trial and categorized them to identify which outcomes researchers have prioritized, with a focus on trials with mortality endpoints.

#### Co- and competing interventions

Including co-interventions in a trial can help to determine whether the clinical benefits that MDA-azithromycin confers persist when other interventions are present. This information can inform whether MDA-azithromycin can supplement or be supplanted by other interventions that may not promote AMR and help to elucidate how MDA-azithromycin reduces mortality. We collected information on interventions that were administered alongside azithromycin with a focus on trials with mortality outcomes. We assessed the presence of competing interventions at the country-level by collecting information on countries’ use of SMC [14]; indicators assessing water, sanitation, and hygiene (WASH) metrics [15]; and indicators assessing immunization status against vaccine-preventable bacterial infections [16]. This information on competing interventions was collected from global databases and publications separate from the clinical trials examined in this review.

#### Spillover effects

When individuals and communities in distinct arms of a clinical trial interact with one another, spillover of treatment effects and both antibiotic-resistant and susceptible bacteria can occur from individuals in one arm to those in the other. Randomization at the community level can help reduce spillover effects between treatment and control groups by ensuring that targeted individuals or households within the same community receive the same intervention. In this analysis, we excluded trials with units of treatment below the community level. We examined the reporting in clinical trials of randomization units, how randomization was done, and how different intervention groups were geographically separated or not to account for spillover effects or “contamination” between trial arms.

#### AMR monitoring

Robust measures to monitor for AMR may be increasingly important as more populations are treated with MDA-azithromycin more frequently. We collected information on how AMR was monitored, including the type of testing (i.e., genotypic versus phenotypic); types of specimens collected; resistance monitored by antibiotic class (e.g., beta-lactam, macrolide); frequency, timing, and duration of testing; and other features, including explanations of AMR in consent forms.

### Gap analysis

Across the five dimensions, we identified clinical trial design features that were common, uncommon, and absent from the trials included in this review. Additionally, we examined whether evidence gaps first published in the WHO guideline had been filled or could be answered with forthcoming evidence from ongoing clinical trials and identified where additional studies were needed.

## Results

**Fig. 2:**
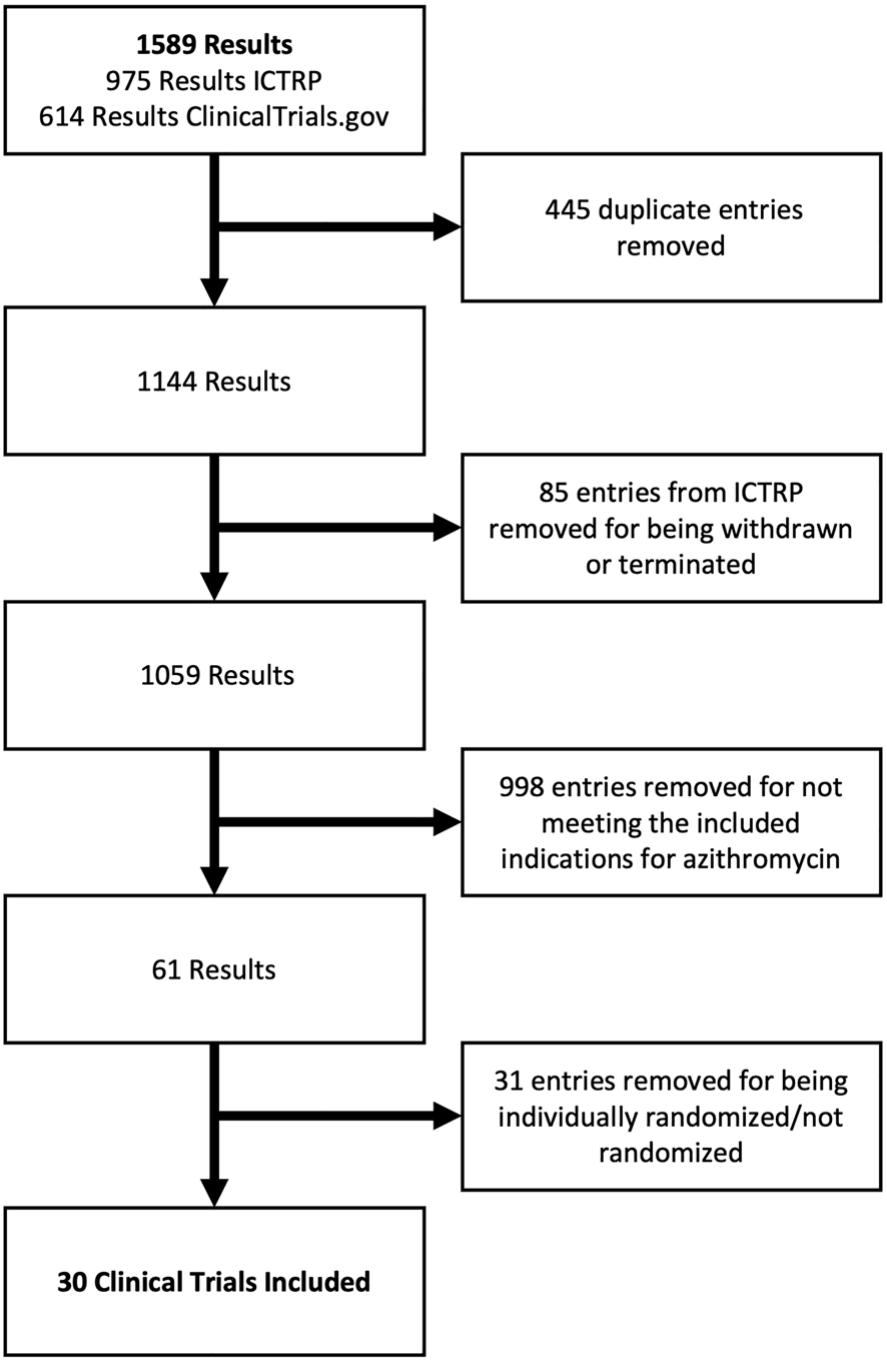
Process of eliminating clinical trials based on exclusion criteria. *ICTRP* International Clinical Trials Registry Platform.

Our initial search on ClinicalTrials.gov and ICTRP identified 1589 studies for further analysis (Fig. 2). We removed 445 duplicate entries between the two registries and a further 85 entries listed in ICTRP that were withdrawn or terminated on ClinicalTrials.gov. Most of the clinical trials excluded (998/1589) from this study failed to meet the inclusion criteria of treating or preventing non-diagnosed diseases or conditions with azithromycin. Half (31/61) of the remaining clinical trials were excluded because they were observational studies or studies for which the intervention was randomized at the individual or household level. A total of 30 clinical trials were included in the analysis.

The subsequent sections discuss the five key areas of clinical trial design in the 30 selected trials.

### Targeting MDA

The 30 clinical trials included in this analysis span 13 countries, with all but four carried out in sub-Saharan Africa (Table 2, S1A/B).

**Table 2:** Geographic location of mass drug administration clinical trials

Geographic Region	Country	Number of clinical trials*	Clinical trial name (Clinical trial ID)	Enrollment status
Sub-Saharan Africa	Ethiopia	9	Stronger SAFE: a community-based cluster-randomized trial to strengthen strategies to eliminate trachoma (ISRCTN40760473)	Completed
			Trachoma Elimination Follow-up (NCT00221364)	Completed
			Trachoma Amelioration in Northern Amhara (TANA; NCT00322972)	Completed
			Tripartite International Research for the Elimination of Trachoma (NCT01202331)	Completed
			Sanitation, Water, and Instruction in Face-washing for Trachoma I/II (NCT02754583)	Ongoing
			Kebele Elimination of Trachoma for Ocular Health (KETFO; NCT03335072)	Ongoing
			Trachoma Elimination Study by Focused Antibiotic (TESFA; NCT03523156)	Suspended (due to security issues)
			Cluster RCT of Co-administration Azithromycin, Albendazole & Ivermectin (NCT03570814)	Completed
			Evaluation of Effect of Stopping Mass Azithromycin Treatment after five years (PACTR201211000437277)	Ongoing
	Niger	9	Impact of Two Alternative Dosing Strategies for Trachoma Control in Niger (NCT00618449)	Completed
			Partnership for Rapid Elimination of Trachoma (NCT00792922)	Completed
			Mortality Reduction After Oral Azithromycin: Mortality Study (NCT02047981)	Completed
			Mortality Reduction After Oral Azithromycin: Morbidity Study (NCT02048007)	Completed
			Mortality Reduction After Oral Azithromycin Contingency: Mortality Study (NCT03338244)	Completed
			Azithromycin Reduction to Reach Elimination of Trachoma (NCT04185402)	Ongoing
			Azithromycin for Child Survival in Niger: Mortality and Resistance Trial (NCT04224987)	Ongoing
			Azithromycin for Child Survival in Niger: Delivery Trial (NCT04774991)	Ongoing
			Azithromycin for Child Survival in Niger: Programmatic Trial (AVENIR; NCT05288023)	Ongoing
	Tanzania	5	Study of Three Alternatives for Mass Treatment in Trachoma Villages of Tanzania (NCT00347607)	Completed
			Partnership for Rapid Elimination of Trachoma (NCT00792922)	Completed
			A Surveillance and Azithromycin Treatment for Newcomers and Travelers Evaluation: The ASANTE Trial (NCT01767506)	Completed
			Mortality Reduction After Oral Azithromycin: Mortality Study (NCT02047981)	Completed
			Mortality Reduction After Oral Azithromycin: Morbidity Study (NCT02048007)	Completed
	Burkina Faso	2	Community Health Azithromycin Trial in Burkina Faso (NCT03676764)	Completed
			Infant Mortality Reduction by the Mass Administration of Azithromycin (NCT04716712)	Ongoing
	Mali	2	Safety of the Co-administration of Three Drugs for Trachoma and Lymphatic Filariasis Elimination (NCT01586169)	Completed
			Effects of Mass Drug Administration of Azithromycin on Mortality and Other Outcomes Among 1–11 Month Old Infants in Mali (NCT04424511)	Ongoing
	Malawi	2	Mortality Reduction After Oral Azithromycin: Mortality Study (NCT02047981)	Completed
			Mortality Reduction After Oral Azithromycin: Morbidity Study (NCT02048007)	Completed
	Benin	1	Periodical Presumptive Treatment for the Control of Gonococcal Infections Among Sex Workers (NCT01329588)	Completed
	Gambia	1	Partnership for Rapid Elimination of Trachoma (NCT00792922)	Completed
	Ghana	1	Periodical Presumptive Treatment for the Control of Gonococcal Infections Among Sex Workers (NCT01329588)	Completed
	South Sudan	1	Enhancing the A in SAFE for Trachoma (NCT05634759)	Completed
Western Pacific	Papua New Guinea	2	Evaluation of an Intensive 3-round MDA Strategy Towards Yaws Eradication (NCT03490123)	Completed
			Safety of Co-administration of IDA and Azithromycin for NTDs (ComboNTDs; NCT03676140)	Completed
	Solomon Islands	1	Azithromycin—Ivermectin Mass Drug Administration for Skin Disease (NCT02775617)	Completed
South Asia	Bangladesh	1	Protein Plus: Improving Infant Growth Through Diet and Enteric Health (NCT03683667)	Completed

^*^Clinical trials taking place in multiple countries are listed for each country

^†^This trial is listed as “Recruiting” on Pan African Clinical Trials Registry, but the anticipated date of last follow-up was May 29, 2015

*MDA* Mass drug administration, *NCT* National clinical trial, *NTD* Neglected tropical disease, *RCT* Randomized controlled trial, *SAFE* Surgery, antibiotics, facial cleanliness, environmental improvement

Only nine of the 30 included trials examined mortality, and all nine trials were conducted in sub-Saharan Africa. These nine trials comprise 11 unique country and time pairings (Table S2). Of these 11 pairings, nine took place in countries where the national under-five mortality rate point estimates exceeded the WHO’s guideline threshold of > 80 deaths per 1000 live births at the time of enrollment, though this guideline was established after several trials had begun or concluded [4]. However, under-five mortality can vary widely within countries, and this guideline did not specify a geographic level (e.g., district-, regional-, or country-level) at which this threshold should apply. For national infant mortality rates, six of these pairings had point estimates that exceeded the WHO guideline threshold of > 60 deaths per 1000 live births [4]. Upon applying the upper-bound estimate for national infant mortality rates, 8 out of the 11 pairings met or exceeded this threshold. Two countries carrying out MDA trials met neither the under-five mortality rate nor the infant mortality rate at the time of enrollment, even when using the upper bound: Malawi and Tanzania in the 2014 MORDOR trial (before the WHO guideline was established).

**Fig. 3:**
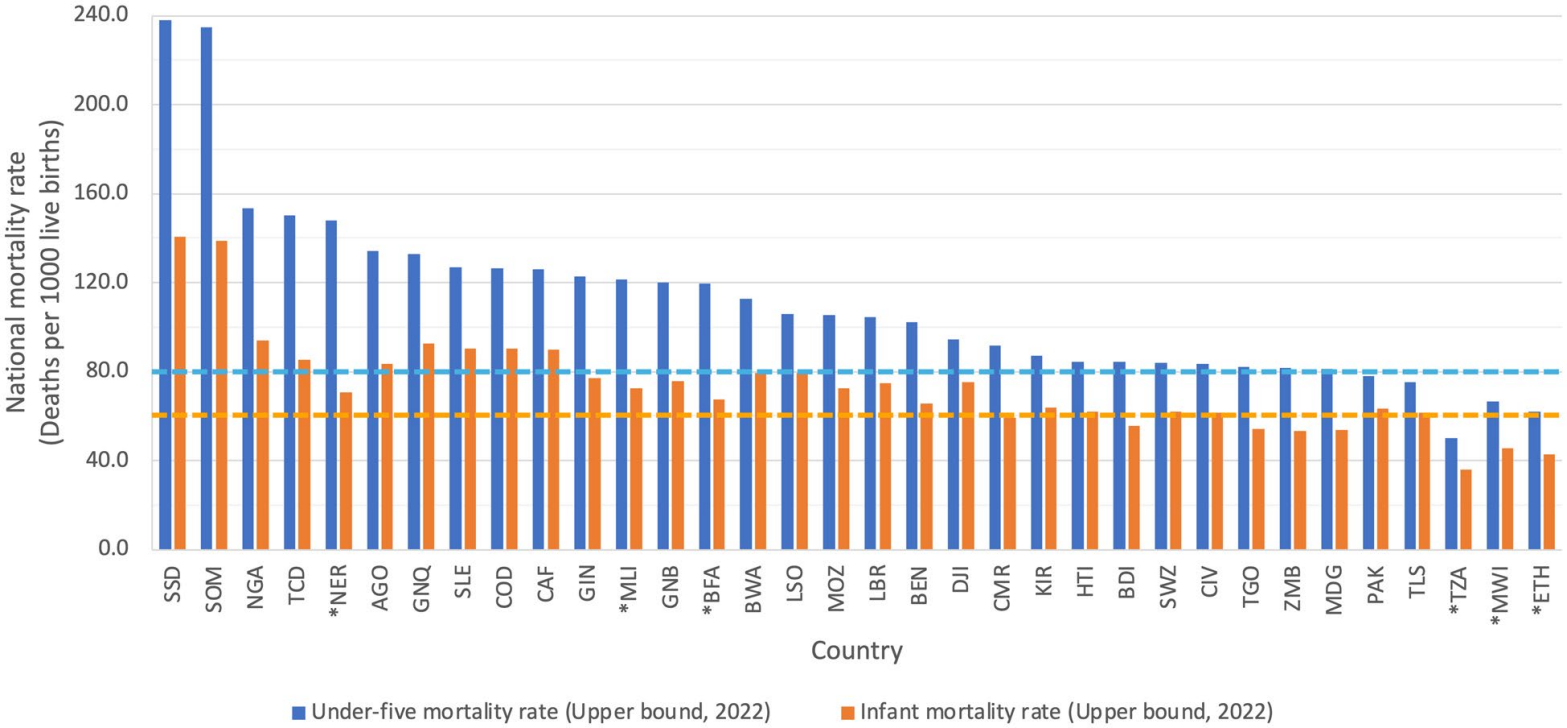
National under-five and infant mortality rates by country (upper bound estimates). The light-blue dashed line shows the WHO guideline recommended under-five mortality rate cutoff, and the light-orange dashed line shows the WHO guideline recommended infant mortality rate cutoff. Countries with an asterisk are those in which the included mortality trials have been conducted or are ongoing. *SSD* South Sudan, *SOM* Somalia, *NGA* Nigeria, *TCD* Chad, *NER* Niger, *AGO* Angola, *GNQ* Equatorial Guinea, *SLE* Sierra Leone, *COD* Democratic Republic of the Congo, *CAF* Central African Republic, *GIN* Guinea, *MLI* Mali, *GNB* Guinea-Bissau, *BFA* Burkina Faso, *BWA* Botswana, *LSO* Lesotho, *MOZ* Mozambique, *LBR* Liberia, *BEN* Benin, *DJI* Djibouti, *CMR* Cameroon, *KIR* Kiribati, *HTI* Haiti, *BDI* Burundi, *SWZ* Eswatini, *CIV* Côte d'Ivoire, *TGO* Togo, *ZMB* Zambia, *MDG* Madagascar, *PAK* Pakistan, *TLS* Timor-Leste, *TZA* United Republic of Tanzania, *MWI* Malawi, *ETH* Ethiopia

Between the WHO guideline publication in August 2020 to the end of 2023, no additional countries had been targeted by randomized community trials investigating MDA-azithromycin to reduce childhood mortality. However, 28 additional countries, both within and outside of sub-Saharan Africa, have under-five and/or infant mortality rates that are higher than the WHO guideline cutoffs but did not have MDA-azithromycin randomized community trials (Fig. 3, Table S3).

None of the nine clinical trials with mortality outcomes include age eligibility criteria below the one-month lower limit recommended by the WHO (Table S4) [4]. However, six of the nine trials target age groups greater than 11 months of age: the upper limit recommended by the WHO [4]. The AVENIR Mortality and Resistance trial randomized communities to three possible treatment arms: (1) all children 1–59 months of age received biannual MDA-azithromycin, (2) children 1–11 months of age received biannual MDA-azithromycin while children 12–59 months received biannual placebo, and (3) all children 1–59 months of age received biannual placebo [17]. The communities in which all children received azithromycin had 14% lower mortality rates for children 1–59 months of age compared to those in which all children received placebo. However, there was not a statistically significant difference in 1–11-month mortality rates for children in communities where only this narrower age group was given azithromycin compared to communities in which all children were given placebo.

### Clinical endpoints

The majority (22/30) of the analyzed clinical trials included primary or secondary endpoints related to the prevalence or incidence of diseases, including those that may have significant morbidity but not mortality (e.g., trachoma and yaws). Our analysis of the nine trials (Table 3) that did have primary or secondary mortality endpoints found that the majority (6/9) of these trials included endpoints that could aid in establishing the putative mechanism for how MDA-azithromycin reduces childhood mortality.

**Table 3:** Included clinical trial studies by endpoint category for the nine community trials with mortality endpoints

	Endpoint category	Example endpoints	Number of studies with endpoint (primary or secondary, *n* = 9)
Endpoints related to putative mechanism	Disease/pathogen prevalence or incidence	Incidence of febrile illness	5*
	Nutrition and development	Length velocity, weight gain, height-for-age Z-score	3
	Cause-specific mortality or hospital admission	Incidence of cause-specific mortality or hospital admission	2
Monitoring of intervention	Markers of resistance	Macrolide resistance (nasal and rectal swabs)	7
	Microbiome	Microbial diversity in nasopharyngeal swab	3
	Drug adverse events	Self-reported and serious adverse events	1
Implementation	Intervention costs/cost-effectiveness	Cost per death averted	4
	Intervention acceptability	Acceptability of the intervention (as assessed by surveys and focus group discussions)	2
	Intervention coverage	Proportion of children reached	2

^*^One trial included in this category solely investigated the prevalence of trachoma, a non-fatal infection

Most of the mortality trials (7/9) included endpoints to measure markers of AMR. Five of the nine trials included endpoints that characterized the prevalence or incidence of one or more diseases (e.g., malaria, incidence of febrile illness) or carriage of pathogenic organisms (e.g., *Campylobacter*). One-third of the trials included nutritional or developmental endpoints. Only two trials included cause-specific reasons for health facility visits or mortality as endpoints. An additional two trials included measures to collect information on cause of death as described in published protocols or analyses [5, 18]. Four of the nine trials examined the costs or cost-effectiveness of the intervention. The characteristics of each of the analyzed clinical trials by endpoint category are reported in Additional File 7.

The nine mortality trials span six countries (Burkina Faso, Ethiopia, Malawi, Mali, Niger, and Tanzania) but took place at different points in time (Table S2). Eight sites had national under-five or infant mortality rates above the currently recommended rates when enrollment began. Seven of the nine sites were associated with community trials for which the effects of azithromycin on childhood mortality are already available: Niger in the 2020 AVENIR mortality and resistance trial; Burkina Faso in the 2019 CHAT trial; Niger in the 2017 MORDORIIMortY5 trial; Niger, Malawi, and Tanzania in the original 2014 MORDOR trial [6]; and Ethiopia in the 2006 TANA trial [5]. There were statistically significant reductions in childhood mortality in three sites: Ethiopia (2006) and Niger (2014, 2020).

In the 2006 TANA trial, researchers observed a 50% lower mortality rate (*P* = 0.01) in children aged 1–9 years in communities given a single dose of azithromycin compared to children in untreated communities [5]. In the MORDOR trial, communities in Malawi, Niger, and Tanzania were randomized to receive biannual MDA-azithromycin or placebo for two years. At the end of the two years, there was a statistically significant reduction of 13.5% in mortality comparing azithromycin to placebo communities when aggregating results from the three countries (95% *CI:* 6.7–19.8, *P* < 0.001), but only the Niger site had a statistically significant reduction on its own (18.1% reduction, 95% *CI:* 10.0–25.5) [6]. In 2014, when the trial began, the estimated baseline under-five and infant mortality rates in Niger were substantially higher than those in Malawi and Tanzania in 2014 (Table S2). A study on specimens stored from the Niger site of the MORDOR trial singled out the presence of IgG antibodies against *Campylobacter* species, compared to other pathogens, as lower in children treated with azithromycin than those receiving placebo, indicating lower force of infection and transmission [19].

Following the conclusion of the original two-year MORDOR trial, the researchers continued to follow consenting communities in Niger and provided one year of biannual MDA-azithromycin to both the azithromycin and placebo communities [20]. At the end of this year (the third in which communities in Niger participated), the researchers did not observe a statistically significant difference in mortality between the original placebo and azithromycin communities (*P* = 0.55) [20]. However, there was a 13.3% reduction (95% *CI:* 5.8%–20.2%, *P* = 0.007) in under-five mortality for the communities that received MDA-azithromycin for the first time compared to the first two years when they received placebo. There was not a significant difference in under-five mortality for the communities that had received a third year of MDA-azithromycin compared to the first two years (95% *CI:* − 12.3–4.5%, *P* = 0.50) [20]. In the MORDORIIMortY5 trial, researchers re-randomized consenting communities to two years of biannual MDA-azithromycin or placebo (i.e., years four and five of participation) but did not observe statistically significant differences in under-five mortality between the treatment arms at the end of the two years (azithromycin 95% *CI:* 23.1–26.5; placebo 95% *CI:* 24.5–28.5) [21].

Researchers for the CHAT trial in Burkina Faso observed an 18% reduction in mortality in the treatment communities compared to placebo communities (incidence rate ratio 95% *CI*: 0.67–1.02, *P* = 0.07) [22]. Although this result was not statistically significant, the researchers noted several reasons why this trial was likely underpowered to detect a difference of this magnitude: sample size calculations were based on higher estimated mortality rates and lower estimated variability between clusters than what the researchers observed, and 27 communities in the azithromycin arm and 30 communities in the placebo arm were excluded due to geopolitical insecurity in the area [22]. Finally, researchers for the 2020 AVENIR mortality and resistance trial reported a 14% lower mortality rate (95% *CI:* 7–22%, *P* < 0.001) when comparing children 1–59 months of age in communities that received biannual MDA-azithromycin to those in placebo communities [17]. However, significant mortality benefits for children 1–11 months of age were observed only in the communities where all children 1–59 months of age were given biannual MDA-azithromycin; these benefits did not occur in communities where only 1–11 month-olds were given this treatment [17].

### Co-interventions and competing interventions

For six of the nine mortality trials, we found information that noted the presence of non-azithromycin interventions with potential mortality-reducing effects (e.g., WASH or routine childhood immunization platforms), either included as a co-intervention in one or more clinical trial arms or mentioned as a competing intervention occurring outside of the clinical trial that could have mortality-reducing effects (e.g., SMC) for the sample population (Table 4).

**Table 4:** Clinical trials assessing mortality with documented co-interventions or competing interventions

Clinical trial	Description of co-intervention or *competing intervention*
Azithromycin for Child Survival in Niger: Programmatic Trial (AVENIR)	Communities in both arms received routine health services provided by community health workers [23]
Azithromycin for Child Survival in Niger: Mortality and Resistance Trial	*Seasonal malaria chemoprevention (amodiaquine/sulfadoxine-pyrimethamine) is given to all children between 3–59 months of age once-monthly during the rainy season. Researchers noted that SMC coverage was at least 80% in study regions during the trial *[17]
Trachoma Amelioration in Northern Amhara (TANA)	Intensive latrine promotion (instruction and resources to build latrines) was provided to half of the communities receiving once-annual MDA-azithromycin [24]
Community Health Azithromycin Trial (CHAT) in Burkina Faso	Targeted azithromycin or placebo is given to children 5–12 of age weeks at their routine vaccine appointment in this trial, nested within a broader community trial [25] *Four rounds of monthly seasonal malaria chemoprevention (amodiaquine/sulfadoxine-pyrimethamine) are administered to children 3–59 months of age between July and October each year*
Infant Mortality Reduction by the Mass Administration of Azithromycin	Azithromycin or placebo was administered through door-to-door Child Health Days programming in which vitamin A and deworming were provided for children 12–59 months of age, with children 6–59 months of age being screened for acute malnutrition [26]
*Effects of Mass Drug Administration (MDA) of Azithromycin on Mortality and Other Outcomes Among 1–11 Month Old Infants in Mali*	*The national health service provides seasonal malaria chemoprevention (amodiaquine/* *sulfadoxine-pyrimethamine) to all children between 3–59 months of age once-monthly between July and October each year *[27]

*MDA* Mass drug administration, *SMC* Seasonal malaria chemoprevention

Across the six countries listed above, access to at least basic drinking water services, at-home handwashing stations, or use of improved latrines and other improved facilities were absent for, on average, nearly one-third to half of the population (Table S5) [15]. About one-fourth of children were estimated to have not received their third dose of pneumococcal conjugate vaccine (PCV), and one-fifth of children were estimated to have incomplete diphtheria-tetanus-pertussis (DTP) immunization status [16]. The trial sites and years for which significant mortality reductions were found (Ethiopia in 2006 and Niger in 2014 and 2020) were in countries that generally had lower proportions of the population benefiting from improved latrines and other improved facilities and of children who had received Bacille Calmette-Guérin (BCG), DTP-1, DTP-3, and the third dose of *Haemophilus influenzae* type B vaccine than those trial sites and years that did not see mortality reductions (Malawi and Tanzania in 2014). In 2014, the estimated percentage of surviving infants who had received the third dose of the PCV vaccine was just 13% in Niger compared to 87% and 93% in Malawi and Tanzania, respectively [16]. Although improved latrine coverage was lacking in Ethiopia and Niger, researchers in the TANA trial found that adding intensive latrine promotion to a single round of MDA-azithromycin provided no additional mortality benefits compared to MDA alone [28] but did provide modest reductions in trachoma prevalence [29].

Seven of the eleven sites are in countries that use SMC, a potential competing intervention in lowering childhood mortality, though coverage of this intervention can vary within a country and has increased in some countries over time [14]. Differences in the distribution and coverage of this intervention between treatment and control arm communities could amplify or attenuate observed differences in mortality rates, depending on where SMC was administered and to what extent. Additionally, if MDA-azithromycin primarily reduces childhood mortality due to antimalarial activity, it is unlikely that communities with high coverage of SMC would benefit from MDA-azithromycin.

In the AVENIR trial, under-five mortality was 14% lower in communities where children 1–59 months of age were given biannual MDA-azithromycin, compared to placebo communities [17]. These mortality benefits were observed despite high coverage of SMC to children 3–59 months of age in the region where these communities were located: this competing intervention had not been prominent in Niger during the MORDOR trial [17]. Researchers in the CHAT trial observed a comparable 18% decrease in mortality in communities receiving biannual MDA-azithromycin compared to biannual placebo [22]. Although this difference was not statistically significant—likely due to underpowering, as noted above—the randomized communities in this trial were in a district in Burkina Faso known to have relatively high SMC implementation and routine childhood vaccine coverage, and universal health coverage for children under five [22].

### Spillover effects

Only four of the thirty included clinical trials, of which one assessed mortality endpoints, described strategies to separate intervention and control communities to reduce potential spillover. Two trials, the TANA and TESFA trials, measured outcomes using sentinel study sites within larger geographic areas in which all villages received the same intervention to reduce the possibility of interactions between communities receiving different interventions [5, 30]. In another trial, communities were selected that were geographically isolated from one another to minimize contamination between arms [31]. The fourth trial ensured that villages were at least 15 km apart [32].

**Fig. 4:**
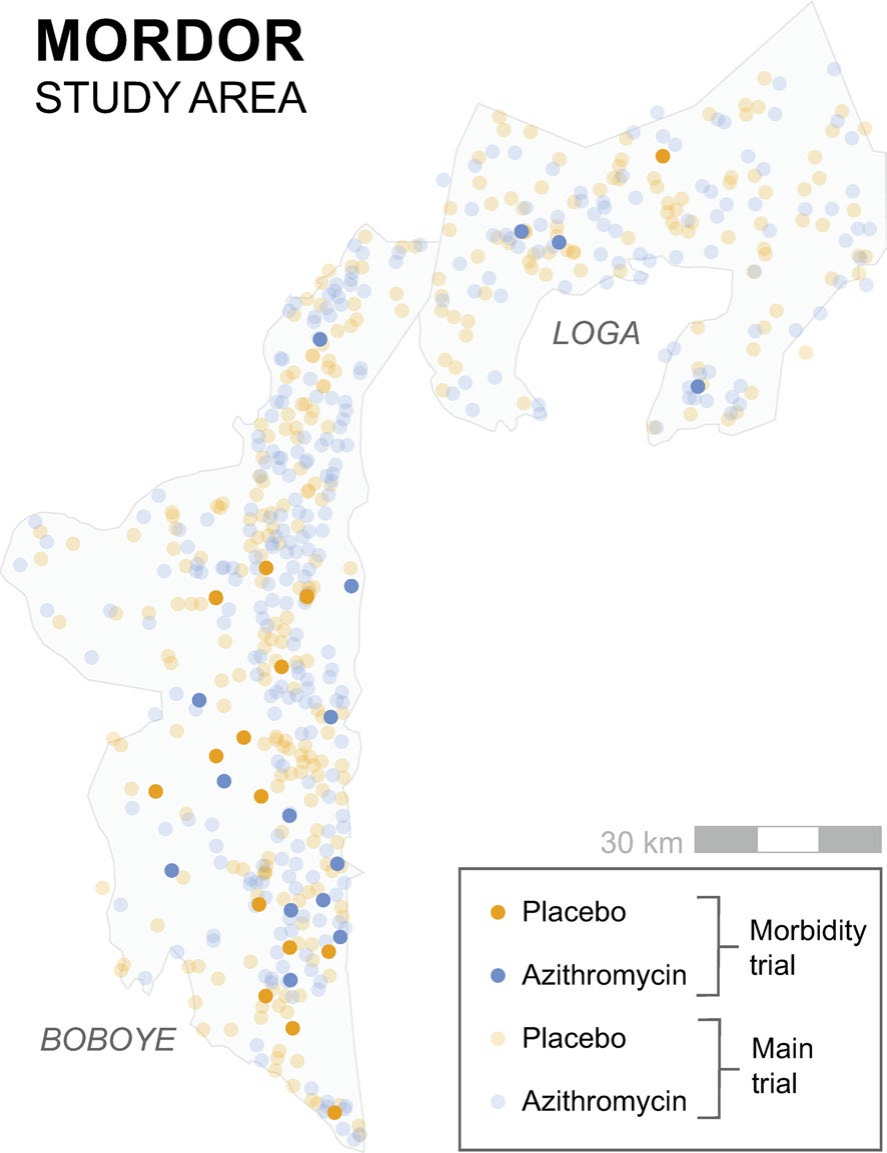
The Niger MORDOR study area. Image from [33]

In the main MORDOR trial, communities were randomized to account for spillover within (i.e., between treated and untreated individuals living in the same community) but not between communities, resulting in several placebo and azithromycin-treated communities existing in close proximity to one another throughout the study (Fig. 4) [33]. A similarly high degree of geographic overlap also appeared to be present in some communities in the Tanzania site of the MORDOR trial, which did not see a statistically significant difference in mortality outcomes [34]. Some geographic overlap between communities with different treatment arms was also present in the CHAT trial [22]. High geographic overlap between treatment and placebo communities could diminish observed differences between communities. For example, untreated communities would likely encounter fewer infected individuals from neighboring, treated communities but might also encounter more individuals carrying drug-resistant bacteria [10].

### AMR monitoring

We found information on plans to monitor for AMR for half (16/30) of the community trials analyzed (Additional File 7) At least one additional trial included plans to monitor for AMR if additional funds were secured [35], and another trial did not provide specifics of monitoring although its parent study did [36]. Details on AMR monitoring for the remainder of the trials were unclear and not found on clinical trial registry listings or in publications.

For the 16 trials that did clearly include AMR monitoring, four applied phenotypic testing (e.g., disk diffusion assays and other tests to detect AMR through in vitro testing), four included genotypic testing (e.g., monitoring for the presence of antibiotic resistance genes associated with phenotypic resistance), and eight included both forms of testing.

Most (14/16) of these trials collected nasopharyngeal swabs to characterize the respiratory microbiome. Two-thirds of the trials (9/16) collected both nasopharyngeal swabs and stool samples or rectal swabs to monitor for AMR in the gut microbiome. The two trials that did not collect either nasopharyngeal or rectal or stool samples focused on yaws and collected only swabs from skin lesions and wounds [37, 38]. While most trials that monitored for AMR also sampled treatment-naïve patients in the control arm, some trials collected specimens from children living in treatment communities but who were too old to be enrolled [27, 39, 40] as well as parents or caregivers [39] in households with eligible children. Researchers for the MORDOR trial found no significant differences in the prevalence of genetic determinants for macrolide, beta-lactam, or tetracycline resistance when comparing nasopharyngeal samples taken from older, untreated children living in treated and untreated communities [41]. These findings held regardless of whether an older child lived in a house with a younger child who had received treatment. In addition to sampling different groups, the AVENIR trial also collected environmental samples for genotypic testing including samples from latrines, water sources, animal feces, and soil [39].

Two-thirds (11/16) of the trials tested for non-macrolide resistance; one trial did not explicitly mention which forms of resistance would be examined, and four trials only reported screening for macrolide resistance. The second most common type of antibiotic resistance monitored for was beta-lactam resistance in at least six trials (primarily for penicillins), followed by tetracycline and clindamycin (five each), and trimethoprim/sulfamethoxazole resistance (four). One trial’s protocol described a “stopping rule” to terminate the trial before a second round of MDA-azithromycin was administered if the prevalence of AMR was deemed to be too high (> 5% macrolide-resistant yaws in the communities randomized to continue MDA compared to the one-round community) [42].

The number of times samples were taken for AMR monitoring differed between trials but generally included baseline and endline sampling. Measuring AMR at these timepoints is critical to consider the impact of AMR on MDA-azithromycin and determine how AMR changes as this intervention is used. An analysis of nasopharyngeal samples taken from participants in the Malawi site of the MORDOR trial revealed high baseline carriage of *Streptococcus pneumoniae* species in placebo and azithromycin communities (85–87%), of which 28% of sample isolates in both groups demonstrated phenotypic resistance to macrolides and 45–47% from these groups demonstrated phenotypic resistance to penicillins [43]. AMR prevalence was markedly lower in the Niger and Tanzania sites, even in treatment communities six months after the fourth round of biannual MDA-azithromycin (12.8% and 13.4%, respectively) [43]. Although the study’s authors noted the possibility that baseline resistance could affect the mortality-reducing effects of MDA-azithromycin, they underscored decreases in mortality with each round of MDA-azithromycin over two years, even as AMR increased in Niger and Malawi.

Most trials (14/16) described inclusion of at least one specimen collection timepoint that occurred after the last dose of azithromycin was given, with some trials collecting specimens as far as 48 months after a final round of MDA was administered [44]. The prevalence of macrolide-resistant *Streptococcus pneumoniae* isolates in children 1–5 years of age living in communities in Ethiopia that had received six rounds of biannual MDA-azithromycin was 76.8%, 30.6%, and 20.8% at six, twelve, and twenty-four months after the final treatment, respectively [45]. Results from the Niger site of the MORDOR trial found that the genetic determinants of macrolide and non-macrolide resistance in bacteria from rectal samples were higher at 36- and 48-months in treated versus placebo communities [46]. When examining phenotypic resistance in bacteria from nasopharyngeal samples at this site, the researchers found a general increase in phenotypic macrolide resistance over 12, 24, and 36 months but no difference in non-macrolide resistance at the 36-month time point [47]. A follow-up study found that the prevalence of genetic determinants of resistance in gut bacteria appeared to increase, then plateau after two to three years of treatment, and differences in non-macrolide resistance were not observed at the 60-month time point [48, 49]. These findings suggest that significant non-macrolide resistance may not develop in response to MDA-azithromycin, and while macrolide resistance can persist for several years, its rise may stabilize over time.

## Discussion

The use of azithromycin—a key first-line antibiotic in the delivery of primary health care in LMICs—in MDA campaigns raises important clinical, policy and ethical considerations. The 2020 WHO guideline that circumscribed its application would have benefited from a stronger evidence base and called for a review in two years of its recommendations. An updated guideline has not yet been published. This review of 30 completed and ongoing clinical trials focused on MDA-azithromycin offers insight on whether that evidence base is coming together, what gaps remain to be filled, and what practices might guide the responsible development of new trials. This review also reflects on the populations and regions that have been targeted, how trials can support a clearer understanding of how this intervention works, the persistence of benefits in settings with competing interventions, and considerations for addressing spillover effects and monitoring AMR.

### MDA-azithromycin trials are geographically concentrated

The WHO guideline development group recommended limiting MDA-azithromycin for childhood mortality to settings similar to those in which mortality benefits were observed, restricting use to high-mortality settings in sub-Saharan Africa [4]. Of the 30 studies analyzed, few were conducted outside of sub-Saharan Africa. All nine mortality trails were conducted in six countries in sub-Saharan Africa despite there being many countries in which, by WHO’s existing recommendations, the use of MDA-azithromycin might be indicated based on infant and under-five mortality rates. Determining whether MDA-azithromycin produces benefits in other high-mortality countries, including those outside sub-Saharan Africa, could prove valuable in informing future iterations of the WHO guideline. Further studies are also needed to determine whether the WHO’s recommended mortality cutoffs for using MDA-azithromycin should be adjusted, and evidence is needed to establish the geographic level (e.g., village, district, region) at which these cutoffs suggest potential for reducing childhood mortality. Detailed analysis of observational studies could support the latter.

### Broader age coverage may be necessary to produce better mortality outcomes

The WHO guideline recommended use of MDA-azithromycin only in children 1–11 months of age, given a small body of evidence suggesting that younger children had the most to gain from this intervention [4, 6]. The AVENIR trial provided compelling evidence that treating a broader age group (1–59 months of age) may be necessary to achieve statistically significant benefits for this narrower range of children [17]. This is likely due to direct benefits, from receiving azithromycin, and indirect benefits, by reducing infections amongst older individuals with whom these children might interact. The benefits that this intervention could have for specific age groups should be considered alongside the risks of wider use. If the results of the AVENIR trial are generalizable to other regions, the treatment of a narrower age range of children, in accordance with current WHO guidelines to limit the overuse of antibiotics, may incur similar risks of AMR in this age group while failing to achieve mortality reductions. At least three trials have examined older age groups (Table S4): findings from these trials could extend the upper 11-month age limit of the WHO’s guideline.

### A variety of trials are needed to clarify relevant mechanisms of action and populations

The exact mechanism by which MDA-azithromycin achieves reductions in mortality is unclear. Azithromycin’s broad antimicrobial activity likely reduces infections and carriage of multiple pathogens that could result in mortality, but the use of a broad-spectrum agent risks AMR in more bacterial species. The effectiveness of MDA-azithromycin may be self-limiting as resistance develops over time. If the mortality-reducing effects of MDA-azithromycin are predominantly linked to a reduction of a subset of the pathogens it acts upon and these pathogens can be identified, targeted alternatives with lower AMR risks, such as synbiotics [50], could be considered instead.

Rolfe et al. reported conflicting results in MDA-azithromycin trials, with some studies finding significant differences in cause-specific mortality and the incidence of infections like malaria, pneumonia, and diarrhea between treated and untreated communities while others found no such differences [11]. The inclusion of endpoints such as cause-specific mortality, the prevalence and incidence of specific communicable diseases, and nutritional and anthropometric health outcomes can help rule in or rule out mechanisms that are likely to improve child survival. However, studies must be sufficiently powered to detect meaningful differences in these endpoints. Few of the analyzed mortality trials included cause-specific mortality or hospitalization and nutritional outcomes.

While individually randomized trials cannot reveal the full effects of azithromycin that occur when administered to an entire community, these trials can provide greater insight as to plausible mechanisms by which MDA-azithromycin directly reduces mortality and who might benefit from receiving this intervention. Several individually randomized trials have been designed to examine the effect of administering azithromycin to specific populations, including healthy pregnant women and/or infants [51–57] and children with acute malnutrition [58, 59]. One completed trial found no differences in mortality, hospitalization, or sick-child visits when azithromycin was administered to infants at well-child visits [60]. In another, administration of a single dose of azithromycin to pregnant women in labor significantly reduced maternal sepsis or death but not stillbirth or neonatal death or sepsis [51]. A third trial found that administering azithromycin to neonates did not lead to improvements in infant growth [61]. However, a subgroup analysis in the cluster-randomized CHAT trial revealed that malnourished children in communities receiving biannual MDA-azithromycin had lower mortality rates than their counterparts in untreated communities [62]. Both individually and cluster-randomized trials are needed to help guide our understanding of the contexts in which implementing MDA-azithromycin, or an alternative intervention, can benefit communities.

### MDA-azithromycin mortality benefits persist in some settings with other interventions

The original WHO guideline included a condition of considering MDA-azithromycin in communities where “existing child survival interventions could be simultaneously strengthened” [4]. The results of the SMCAZ trial had raised concerns that mortality-reducing effects of MDA-azithromycin might be less pronounced in communities with high coverage of other interventions such as childhood immunizations and SMC [7]. However, this trial’s intervention was not randomized at the household level and combined mortality and hospital admission into one endpoint. Findings from the community-randomized AVENIR and CHAT trials suggest that MDA-azithromycin can still produce childhood mortality benefits in settings with high coverage of SMC and other child health services including immunization [17, 22]. Evidence from other settings could support greater understanding of whether any competing interventions, alone or in combination, might preclude the need for MDA-azithromycin in high-mortality settings.

While strengthening SMC and immunizations alongside MDA-azithromycin could have additive effects on mortality, results from the TANA trial suggest that adding intensive latrine promotion to MDA-azithromycin provides benefits for trachoma prevalence [29] but not mortality [28]. Conversely, promoting facial cleanliness and providing environmental improvements without MDA-azithromycin may not be sufficient to achieve trachoma elimination in settings with hyperendemic trachoma [63]. However, focusing solely on mortality reduction may exclude interventions that complement MDA-azithromycin in other ways. WASH interventions could help to control the spread of AMR associated with MDA-azithromycin [64]. Integration of MDA-azithromycin into existing health platforms such as Child Health Days and vaccination programs could simplify administration and improve program cost-effectiveness.

### More evidence on the necessary frequency and duration of MDA-azithromycin is needed

The WHO recommended biannual administration of MDA-Azithromycin to balance the benefits and risks of repeated exposure to azithromycin [4]. One completed [5] and one ongoing [27] trial have sought to examine the effects of quarterly MDA-azithromycin on childhood mortality. Furthermore, the duration of biannual MDA-azithromycin necessary to elicit mortality benefits and persistence of these benefits after MDA-azithromycin ends are unclear. Continuing studies of MORDOR communities in Niger suggest similar mortality rates in communities receiving biannual MDA-azithromycin for one year compared to those that had received the intervention for three years [20]. Additionally, when researchers re-randomized communities that had received one to three years of biannual MDA-azithromycin to biannual MDA-azithromycin or placebo, they found no differences in under-five mortality between the treatment arms [21]. The persistence of mortality benefits for two years after a single year of biannual MDA-azithromycin could suggest the value of occasional, rather than indefinite, biannual MDA-azithromycin campaigns. Such an approach could produce short-term mortality benefits while allowing time for AMR to wane in a community before the next campaign.

### Spillover and AMR monitoring efforts in trials could be strengthened

Two notable research gaps highlighted by the WHO Guideline Development Group included the circulation of resistant bacteria beyond treated children and the long-term effects of MDA-azithromycin use on AMR [4]. Some trials, including several non-mortality trials, attempted to minimize AMR and other spillover effects by accounting for the geographic location of communities [5, 30–32]. While some treatment and control communities in the MORDOR trial appeared to have a high degree of geographic proximity to one another, the observed difference in mortality between these communities was sufficiently high to achieve statistical significance [6]. However, spillover effects between clinical trial arms could diminish observed differences in benefits and risks, potentially leading to an underpowered study in other settings. Careful consideration and planning of clinical trials can limit these effects and provide critical data necessary to establish mathematical models to better predict the impact of these effects [65]. Additional studies to quantify spillover effects within communities—as illustrated by the AVENIR trial—are also needed [17].

Current evidence on the effects of MDA-azithromycin on AMR suggests that macrolide resistance remains elevated when MDA campaigns occur biannually, but the effects of these campaigns on non-macrolide resistance have been mixed [46–48]. It is likely that longer periods without biannual MDA-azithromycin are necessary before the heightened AMR recedes [45, 48]. Many, but not all, trials that included AMR monitoring details collected multiple specimen types, screened for non-macrolide resistance, or conducted both phenotypic and genotypic resistance testing. Increased efforts to ensure that trials incorporate these characteristics would provide valuable information on the potential long-term effects of MDA-azithromycin on AMR [66].

Studies from the Niger site of the MORDOR trial suggest minimal or transient AMR spillover effects to older children living in the same communities or households as treated children [41]. Additional studies to examine the effects of MDA-azithromycin on carriage of AMR bacteria by others living in the same homes or communities as treated children, including caregivers, and the effects of MDA-azithromycin on producing environmental AMR reservoirs could prove valuable.

Monitoring AMR before, during, and well after MDA-azithromycin can help to characterize the changing incidence and extent of AMR in MDA communities, how resistance affects the effectiveness of MDA-azithromycin, and how quickly AMR wanes in communities that have ceased MDA. Interim measurements can enable implementation of AMR stopping rules while storage of biospecimens could enable future analyses relevant to identifying MDA-azithromycin’s mechanism of action and effects on AMR and microbial diversity [19]. Characterization of local antibiotic prescribing and usage trends and AMR to commonly used antibiotics can contextualize results while describing AMR in informed consent forms can uphold community autonomy in understanding risks [67]. These measures were included in the ongoing LAKANA trial [27] and may be worth considering in future MDA-azithromycin trials and programs.

### Future trends in MDA-azithromycin

As the use of azithromycin to promote better health outcomes is further investigated or implemented, the magnitude and frequency of azithromycin use from both traditional MDA and targeted subpopulation approaches could have important implications. As trachoma is controlled or eliminated in more districts, and trachoma-targeted MDA programs are concluded, incidental benefits could end as well. Unlike MDA-azithromycin for childhood mortality, these campaigns include administration to the entire community, regardless of age, thus the volume of azithromycin used for childhood mortality campaigns is likely to be much lower in comparison. Even so, the broad use of azithromycin for more age groups and conditions, such as malnutrition, could lead to increased azithromycin use in some populations.

The WHO guidelines on MDA-azithromycin to reduce childhood mortality balanced the potential mortality-reducing benefits of this intervention against the risks of increased AMR in treated communities. In doing so, a narrow age range and thresholds for age-specific mortality in these settings to guide consideration of MDA-azithromycin were established. These circumspect guidelines were originally based upon just three trials, of which two were community-based, cluster-randomized trials. Since then, the number of mortality trials with resultant findings have increased, with some results suggesting the need for a broader age range and others underscoring uncertainty regarding what communities may benefit when different competing interventions are present. Further trials should be designed to fill these identified gaps in evidence and work to build collectively towards a shared understanding of how to update the 2020 WHO recommendations on MDA-azithromycin use.

## Limitations

There are several important limitations to note in this analysis. This study sought to find all relevant information related to the analyzed trials, but the availability of information, including published protocols, varied between trials. The absence of publicly available information does not necessarily mean that certain practices such as AMR monitoring or geographic separation of trial sites were not conducted, nor that secondary analyses will not be conducted in the future. Furthermore, clinical trial protocols are not static and may be amended given shifts in resources and logistical challenges. These limitations underscore the value of greater transparency in both clinical trial registries and publications.

Non-randomized studies were excluded; these studies could provide information on the frequency of practices like AMR monitoring and temporal trends in clinical outcomes. Additionally, biannual MDA-azithromycin to reduce childhood mortality could already be underway in some settings outside of clinical trials. The analysis of competing interventions and underlying characteristics for study countries here used data at the country level. Although these data provide estimates of key characteristics like mortality rates and immunization coverage, sub-national regions may vary dramatically across these measures. Upper-bound mortality rate estimates allowed for a more conservative approach for inclusion, but the use of data at the subnational level could improve this analysis. Finally, mortality rate estimates themselves change over time: the availability and inclusion of additional data have led to substantial changes in mortality rate estimates published by the UN IGME for some countries, including Niger between 2021 and 2022 [68, 69]. Relying on strict mortality cutoffs for the inclusion or exclusion of settings for treatment with MDA-azithromycin at a given point in time may arbitrarily include or exclude settings.

## Conclusions

The analysis of community trials using MDA-azithromycin provides valuable information on useful principles guiding clinical trial design, progress toward addressing research gaps, and areas for further investigation. Ongoing clinical trials have the potential to fill some gaps in evidence needed to revisit and update the WHO’s guideline on MDA-azithromycin to reduce childhood mortality. With the need for additional evidence also comes the need to ensure that trials are appropriately designed to adequately assess benefits and risks. However, instituting good practices identified in mortality and non-mortality MDA-azithromycin trials, would be needed. These might include long-term monitoring of mortality benefits and additional endpoints, expansion of AMR monitoring programs to include phenotypic and genotypic testing, and reducing spillover. However, there are trade-offs with the greater resource intensity required to undertake these efforts.

As the WHO looks to revise its guidelines, studies have been completed or are underway to answer key research questions regarding the long-term effects of MDA-azithromycin on AMR, which age groups can benefit, and implementation costs. Forthcoming results are likely to shed light on the impacts of increasing the frequency of MDA-azithromycin, the extent to which AMR may spill over to untreated community members, and the effects of integrating MDA-azithromycin as part of scaling other community interventions. Results from one of the trials included in this analysis suggest that expansion of the recommended 1–11 month age group may be necessary to realize the benefits of MDA-azithromycin. The effectiveness of MDA-azithromycin in high-mortality countries outside sub-Saharan Africa remains unknown. Little information is available on how long risks and benefits may persist in different settings after biannual MDA-azithromycin concludes, which competing interventions may complement or mitigate the need for MDA-azithromycin, and how generalizable findings in one setting are to others. Concerted efforts to answer these questions through careful study design and timely publication of results are necessary. Nevertheless, the evidence that has been produced since 2020 warrants the development of updated WHO guidelines and a roadmap for gap-filling research and regular revision as new evidence emerges.

## Supplementary Information

Additional file 1. Total estimated number of participants across the 30 clinical trials by region.

Additional file 2. Estimated number of participants for each of the 30 clinical trials by region.

Additional file 3. Mass drug administration clinical trials with mortality endpoints.

Additional file 4. Under-five and infant mortality rates by country (2022).

Additional file 5. Age eligibility requirements and sub-groups assessed for clinical trials with mortality outcomes.

Additional file 6. National characteristics of community trial sites.

Additional file 7. Endpoint and antimicrobial resistance monitoring information for the analyzed trials. The presented endpoint categories are non-exhaustive and reflect major categories of endpoints identified in this analysis.

## Data Availability

The datasets generated during the current study are available from the corresponding author on reasonable request.

## Abbreviations

AMR: Antimicrobial resistance
A-PLUS: Azithromycin-Prevention in Labor Use Study
AVENIR: Azithromycin for Child Survival in Niger
AWaRe: Access, Watch, Reserve
BCG: Bacille Calmette-Guérin
CHAT: Community Health Azithromycin Trial
COVID-19: Coronavirus disease 2019
DTP: Diphtheria-Tetanus-Pertussis
ICTRP: International Clinical Trials Registry Platform
LAKANA: Large-scale Assessment of the Key health-promoting Activities of two New MDA regimens with Azithromycin
LMICs: Low- and middle-income countries
MDA: Mass drug administration
MORDOR: Macrolides Oraux pour Réduire les Décès avec un Oeil sur la Résistance
NCT: National clinical trial
NTD: Neglected tropical disease
PCV: Pneumococcal conjugate vaccine
UN IGME: United Nations Inter-agency Group for Child Mortality Estimation
RCT: Randomized controlled trial
SAFE: Surgery, antibiotics, facial cleanliness, environmental improvement
SMCAZ: Seasonal Malaria Chemoprevention plus Azithromycin in African children
SMC: Seasonal malaria chemoprevention
TANA: Trachoma Amelioration in Northern Amhara
TESFA: Trachoma Elimination Study by Focused Antibiotic
WASH: Water, sanitation, and hygiene
WHO: World Health Organization
