# Supplementary material for: Mass drug administration trials of azithromycin: an analysis to inform future research and guidelines
Source: Infect Dis Poverty. 2025 Jul 21;14:73. doi: 10.1186/s40249-025-01322-8 (PMC12278655; doi:10.1186/s40249-025-01322-8)
Supplement: Supplementary file 1 — Additional file 1. Total estimated number of participants across the 30 clinical trials by region. [file 40249_2025_1322_MOESM1_ESM.pdf]

Supplementary Table 1A – Total estimated number of participants across the 30 clinical trials by region

**Geographic region**	**Number of clinical trials**	**Estimated number of participants (Including control)**
Sub-Saharan Africa	26	4,028,437
Western Pacific	3	77,967
South Asia	1	5283
**Total**	30	4,111,687
