# Supplementary material for: Mass drug administration trials of azithromycin: an analysis to inform future research and guidelines
Source: Infect Dis Poverty. 2025 Jul 21;14:73. doi: 10.1186/s40249-025-01322-8 (PMC12278655; doi:10.1186/s40249-025-01322-8)
Supplement: Supplementary file 2 — Additional file 2. Estimated number of participants for each of the 30 clinical trials by region. [file 40249_2025_1322_MOESM2_ESM.pdf]

Supplementary Table 1B – Estimated number of participants for each of the 30 clinical trials by region

Region (Total number of participants)	Clinical trial name (Clinical trial ID)	Enrollment year	Country/ countries	Number of participants
Sub-Saharan Africa (4,028,437)	Periodical Presumptive Treatment for the Control of Gonococcal Infections Among Sex Workers (NCT01329588)	2001	Benin, Ghana	636
	Community Health Azithromycin Trial in Burkina Faso (NCT03676764)	2018	Burkina Faso	447 780
	Infant Mortality Reduction by the Mass Administration of Azithromycin (NCT04716712)	2021	Burkina Faso	694 400
	Trachoma Elimination Follow-up (NCT00221364)	2005	Ethiopia	20 000
	Trachoma Amelioration in Northern Amhara (TANA) (NCT00322972)	2006	Ethiopia	33 000
	Tripartite International Research for the Elimination of Trachoma (NCT01202331)	2010	Ethiopia	29 000
	Evaluation of Effect of Stopping Mass Azithromycin Treatment after five years (PACTR201211000437277)	2012	Ethiopia	40 500
	Sanitation, Water, and Instruction in Face-washing for Trachoma I/II (NCT02754583)	2016	Ethiopia	220 000
	Kebele Elimination of Trachoma for Ocular Health (KETFO) (NCT03335072)	2017	Ethiopia	320 000
	Trachoma Elimination Study by Focused Antibiotic (TESFA) (NCT03523156)	2018	Ethiopia	53 384
	Cluster RCT of Co-administration Azithromycin, Albendazole & Ivermectin (NCT03570814)	2018	Ethiopia	13 511
	Stronger SAFE: a community-based cluster-randomized trial to strengthen strategies to eliminate trachoma (ISRCTN40760473)	2021	Ethiopia	44 200
	Mortality Reduction After Oral Azithromycin: Mortality Study (NCT02047981)	2014	Malawi, Niger, Tanzania	190 238
	Mortality Reduction After Oral Azithromycin: Morbidity Study (NCT02048007)	2014	Malawi, Niger, Tanzania	72 000
	Safety of the Co-administration of Three Drugs for Trachoma and Lymphatic Filariasis Elimination (NCT01586169)	2012	Mali	3 011
	Effects of Mass Drug Administration of Azithromycin on Mortality and Other Outcomes Among 1–11 Month Old Infants in Mali (NCT04424511)	2020	Mali	100 000

	Impact of Two Alternative Dosing Strategies for Trachoma Control in Niger (NCT00618449)	2008	Niger	1 139
	Partnership for Rapid Elimination of Trachoma (NCT00792922)	2008	Niger, Tanzania, Gambia	98 977
	Mortality Reduction After Oral Azithromycin Contingency: Mortality Study (NCT03338244)	2017	Niger	66 228
	Azithromycin Reduction to Reach Elimination of Trachoma (NCT04185402)	2019	Niger	100 000
	Azithromycin for Child Survival in Niger: Mortality and Resistance Trial (NCT04224987)	2020	Niger	1 106 050
	Azithromycin for Child Survival in Niger: Delivery Trial (NCT04774991)	2021	Niger	12 057
	Azithromycin for Child Survival in Niger: Programmatic Trial (AVENIR) (NCT05288023)	2022	Niger	250 000
	Enhancing the A in SAFE for Trachoma (NCT05634759)	2022	South Sudan	29 000
	A Surveillance and Azithromycin Treatment for Newcomers and Travelers Evaluation: The ASANTE Trial (NCT01767506)	2013	Tanzania	80 626
	Study of Three Alternatives for Mass Treatment in Trachoma Villages of Tanzania (NCT00347607)	2006	Tanzania	2 700
Western Pacific	Safety of Co-administration of IDA and Azithromycin for NTDs (ComboNTDs, NCT03676140)	2018	Papua New Guinea	20 000
	Evaluation of an Intensive 3-round MDA Strategy Towards Yaws Eradication (NCT03490123)	2018	Papua New Guinea	56 676
	Azithromycin – Ivermectin Mass Drug Administration for Skin Disease (NCT02775617)	2016	Solomon Islands	1 291
Asia	Protein Plus: Improving Infant Growth Through Diet and Enteric Health (NCT03683667)	2018	Bangladesh	5 283
