# Supplementary material for: Mass drug administration trials of azithromycin: an analysis to inform future research and guidelines
Source: Infect Dis Poverty. 2025 Jul 21;14:73. doi: 10.1186/s40249-025-01322-8 (PMC12278655; doi:10.1186/s40249-025-01322-8)
Supplement: Supplementary file 3 — Additional file 3. Mass drug administration clinical trials with mortality endpoints. [file 40249_2025_1322_MOESM3_ESM.pdf]

Supplementary Table 2 –Mass drug administration (MDA) clinical trials with mortality endpoints.

**Clinical trial name (Clinical trial ID)**	**Country**	**Enrollment year**	**Under-five mortality rate (Point estimate)**	**Under-five mortality rate (Upper bound)**	**Infant mortality rate (Point estimate)**	**Infant mortality rate (Upper bound)**	**Mortality results**
Trachoma Amelioration in Northern Amhara (TANA, NCT00322972)	Ethiopia	2006	**103.3**	**111.3**	**66.4**	**70.9**	Mortality odds ratio comparing treatment to control group: 0.51 (95% *CI*: 0.29–0.90, $P = 0.02$) [1]
Tripartite International Research for the Elimination of Trachoma (TIRET [TANA II] NCT01202331)	Ethiopia	2010	**82.5**	**89.9**	54.6	58.8	Not available
Mortality Reduction After Oral Azithromycin: Mortality Study (MORDORI Mort, NCT02047981)	Niger	2014	**127.8**	**142.9**	**63.9**	**69.2**	Under-five mortality rate 18.1% lower (95% *CI*: 10.0–25.5, $P < 0.001$) in azithromycin versus placebo group<br><br>Aggregate of Malawi, Niger, and Tanzania: under-five mortality rate 13.5% lower (95% *CI*: 6.7–19.8, $P < 0.001$) in azithromycin versus placebo group [2]

Mortality Reduction After Oral Azithromycin: Mortality Study (MORDORI Mort, NCT02047981)	Malawi	2014	59.6	67.2	40.3	44.4	Under-five mortality rate 5.7% lower (95% *CI*: -9.7–18.9, *P* = 0.45) in azithromycin versus placebo group<br><br>Aggregate of Malawi, Niger, and Tanzania: under-five mortality rate 13.5% lower (95% *CI*: 6.7–19.8, *P* < 0.001) in azithromycin versus placebo group [2]
Mortality Reduction After Oral Azithromycin: Mortality Study (MORDORI Mort, NCT02047981)	Tanzania	2014	56.7	62.5	40.1	43.4	Under-five mortality rate 3.4% lower (95% *CI*: -21.2–23.0, *P* = 0.77) in azithromycin versus placebo group<br><br>Aggregate of Malawi, Niger, and Tanzania: under-five mortality rate 13.5% lower (95% *CI*: 6.7–19.8, *P* < 0.001) in azithromycin versus placebo group [2]
Mortality Reduction After Oral Azithromycin Contingency: Mortality Study (NCT03338244)	Niger	2017	**124.3**	**141.7**	**62.7**	**68.8**	Positive, significant in communities receiving MDA-azithromycin for the first time
Community Health Azithromycin Trial in Burkina Faso (CHAT, NCT03676764)	Burkina Faso	2019	**87.3**	**119.5**	53.8	**67.3**	Mortality incidence rate ratio 0.82 (95% *CI*: 0.67–1.02, *P* = 0.07) in azithromycin versus placebo group [3]

Effects of Mass Drug Administration of Azithromycin on Mortality and Other Outcomes Among 1-11 Month Old Infants in Mali (LAKANA, NCT04424511)	Mali	2020	**99.7**	**122.1**	**62.9**	**73.1**	Not available
Azithromycin for Child Survival in Niger: Mortality and Resistance Trial (AVENIR, NCT04224987)	Niger	2020	**121.0**	**144.1**	**61.6**	**69.6**	Under-five mortality 14% lower (95% *CI*: 7–22, *P* < 0.001) in azithromycin versus placebo groups [4]
Infant Mortality Reduction by the Mass Administration of Azithromycin (MIRAMA, NCT04716712)	Burkina Faso	2021	**81.4**	**119.4**	51.2	**67.3**	Not available
Azithromycin for Child Survival in Niger: Programmatic Trial (AVENIR Programmatic Trial, NCT05288023)	Niger	2022	**117.3**	**147.7**	**60.3**	**70.8**	Not available

Bolded numbers indicate values which are above the designated WHO threshold (under-five mortality rates > 80 deaths per 1000 live births or infant mortality rates > 60 deaths per 1000 live births). *Estimated national under-five and infant mortality rates were sourced from the 2023 UN IGME *Levels and Trends in Child Mortality* report for each year in which enrollment began [5].

[1] Porco TC, Gebre T, Ayele B, House J, Keenan J, Zhou Z, et al. Effect of mass distribution of azithromycin for trachoma control on overall mortality in Ethiopian children: a randomized trial. JAMA 2009;302:962–8. https://doi.org/10.1001/jama.2009.1266.
[2] Keenan JD, Bailey RL, West SK, Arzika AM, Hart J, Weaver J, et al. Azithromycin to Reduce Childhood Mortality in Sub-Saharan Africa. N Engl J Med 2018;378:1583–92. https://doi.org/10.1056/NEJMoa1715474.
[3] Oldenburg CE, Ouattara M, Bountogo M, Boudo V, Ouedraogo T, Compaoré G, et al. Mass Azithromycin Distribution to Prevent Child Mortality in Burkina Faso: The CHAT Randomized Clinical Trial. JAMA 2024;331:482. https://doi.org/10.1001/jama.2023.27393.
[4] O'Brien KS, Arzika AM, Amza A, Maliki R, Aichatou B, Bello IM, et al. Azithromycin to Reduce Mortality — An Adaptive Cluster-Randomized Trial. N Engl J Med 2024. https://doi.org/10.1056/NEJMoa2312093.
[5] United Nations Inter-agency Group for Child Mortality Estimation (UN IGME). Levels & Trends in Child Mortality Report 2023. United Nations Children's Fund (UNICEF); 2024.
