# Supplementary material for: Mass drug administration trials of azithromycin: an analysis to inform future research and guidelines
Source: Infect Dis Poverty. 2025 Jul 21;14:73. doi: 10.1186/s40249-025-01322-8 (PMC12278655; doi:10.1186/s40249-025-01322-8)
Supplement: Supplementary file 4 — Additional file 4. Under-five and infant mortality rates by country (2022). [file 40249_2025_1322_MOESM4_ESM.pdf]

Supplementary Table 3 – Under-five and infant mortality rates by country (2022).

Country	Under-five mortality rate (Point estimate, 2022)	Under-five mortality rate (Upper bound, 2022)	Infant mortality rate (Point estimate, 2022)	Infant mortality rate (Upper bound, 2022)	Previous/ ongoing mortality trial
South Sudan	**98.8**	**238.1**	**63.8**	**140.7**	
Somalia	**106.1**	**235.0**	**68.0**	**138.9**	
Nigeria	**107.2**	**153.5**	**68.5**	**94.1**	
Chad	**102.9**	**150.0**	**64.1**	**85.1**	
Niger	**117.3**	**147.7**	**60.3**	**70.8**	X
Angola	66.9	**134.3**	45.7	**83.6**	
Equatorial Guinea	73.4	**132.9**	55.1	**92.8**	
Sierra Leone	**100.8**	**126.9**	**76.0**	**90.5**	
Democratic Republic of the Congo	75.6	**126.6**	**60.1**	**90.3**	
Central African Republic	**96.8**	**125.9**	**73.5**	**90.1**	
Guinea	**96.0**	**122.8**	**62.2**	**77.3**	
Mali	**93.8**	**121.3**	**60.1**	**72.7**	X
Guinea-Bissau	71.9	**120.2**	48.6	**75.9**	
Burkina Faso	78.8	**119.5**	50.1	**67.3**	X
Botswana	38.7	**113.0**	31.2	**79.3**	
Lesotho	72.2	**106.0**	56.4	**79.4**	
Mozambique	66.2	**105.4**	48.7	**72.7**	
Liberia	73.2	**104.5**	54.9	**74.9**	
Benin	**80.8**	**102.1**	53.6	**65.7**	
Djibouti	51.9	**94.2**	44.1	**75.2**	
Cameroon	69.8	**91.6**	47.0	59.1	
Kiribati	56.4	**87.2**	43.9	**63.8**	
Haiti	56.5	**84.2**	44.0	**61.9**	
Burundi	50.5	**84.2**	36.4	55.6	
Eswatini	50.0	**83.8**	39.7	**62.0**	
Côte d'Ivoire	69.4	**83.4**	52.4	**61.3**	
Togo	60.4	**81.9**	42.1	54.2	
Zambia	55.6	**81.5**	39.0	53.4	
Madagascar	65.8	**81.0**	45.1	53.8	
Pakistan	61.0	78.0	51.0	**63.5**	
Timor-Leste	48.6	75.4	41.5	**61.7**	

United Republic of Tanzania	40.5	50.0	30.0	35.7	X
Malawi	40.1	66.7	30.1	45.5	X
Ethiopia	46.2	61.8	33.9	42.9	X

Countries in which mortality trials using azithromycin have been conducted or are currently being conducted are included in addition to countries which meet the WHO guideline's criterion for consideration of mass drug administration of azithromycin to reduce childhood mortality (under-five mortality rates > 80 deaths per 1000 live births or infant mortality rates > 60 deaths per 1000 live births). **Bolded** numbers indicate values which are above the designated WHO threshold. Data from 2023 UN IGME [1]

[1] United Nations Inter-agency Group for Child Mortality Estimation (UN IGME). Levels & Trends in Child Mortality Report 2023. United Nations Children's Fund (UNICEF); 2024.
