# Supplementary material for: Mass drug administration trials of azithromycin: an analysis to inform future research and guidelines
Source: Infect Dis Poverty. 2025 Jul 21;14:73. doi: 10.1186/s40249-025-01322-8 (PMC12278655; doi:10.1186/s40249-025-01322-8)
Supplement: Supplementary file 5 — Additional file 5. Age eligibility requirements and sub-groups assessed for clinical trials with mortality outcomes. [file 40249_2025_1322_MOESM5_ESM.pdf]

Supplementary Table 4– Age eligibility requirements and sub-groups assessed for clinical trials with mortality outcomes

Clinical trial name (Clinical trial ID)	Ages eligible for azithromycin in study	Childhood mortality age groups analyzed
Trachoma Amelioration in Northern Amhara (TANA; NCT00322972)	12 months and older	1–5 years<br>6–10 years<br>Total childhood mortality (≥ 1 year of age)
Tripartite International Research for the Elimination of Trachoma (NCT01202331)	6 months and older	6 months to 5 years<br>6–10 years<br>> 10 years
Mortality Reduction After Oral Azithromycin: Mortality Study (NCT02047981)	1–60 months	1–5 months<br>6–11 months<br>12–23 months<br>24–59 months
Mortality Reduction After Oral Azithromycin Contingency: Mortality Study (NCT03338244)	1–60 months	1–5 months<br>6–11 months<br>12–23 months<br>24–59 months
Community Health Azithromycin Trial in Burkina Faso (NCT03676764)	1–59 months	4–12 weeks<br>1–59 months
Azithromycin for Child Survival in Niger: Mortality and Resistance Trial (NCT04224987)	1–59 months	1–11 months<br>12–59 months
Effects of Mass Drug Administration of Azithromycin on Mortality and Other Outcomes Among 1-11 Month Old Infants in Mali (NCT04424511)	1–11 months	1–5 months<br>6–11 months
Infant Mortality Reduction by the Mass Administration of Azithromycin (NCT04716712)	1–11 months	1–11 months
Azithromycin for Child Survival in Niger: Programmatic Trial (AVENIR, NCT05288023)	1–11 months	Not listed
