# Supplementary material for: Mass drug administration trials of azithromycin: an analysis to inform future research and guidelines
Source: Infect Dis Poverty. 2025 Jul 21;14:73. doi: 10.1186/s40249-025-01322-8 (PMC12278655; doi:10.1186/s40249-025-01322-8)
Supplement: Supplementary file 6 — Additional file 6. National characteristics of community trial sites. [file 40249_2025_1322_MOESM6_ESM.pdf]

Supplementary Table 5— National characteristics of community trial sites

**Community trial (Enrollment year)**	**Country**	**U5 mortality point estimate (Upper bound)**	**Infant mortality point estimate (Upper bound)**	**% of population using at least basic drinking water services**	**% of population with no hand-washing facility at home**	**% of population using improved latrines and other improved facilities**	**% of live births who received BCG**	**% of surviving infants who received DTP-1**	**% of surviving infants who received DTP-3**	**% of surviving infants who received the third dose of Hib-containing vaccine**	**% of surviving infants who received the third dose of PCV**	**SMC is used (2022)**	**Trachoma interventions needed?**
TANA (2006)	Ethiopia	**103.3 (111.3)**	**66.4 (70.9)**	27.8	NA	7.6	66	64	46	NA	NA	No	Yes
TIRET (TANA II, 2010)	Ethiopia	**82.5 (89.9)**	54.6 (58.8)	34.0	39.1	9.4	76	77	61	62	NA	No	Yes
MORDORIMort (2014)	Niger	**127.8 (142.9)**	**63.9 (69.2)**	45.4	22.9	15.2	87	95	81	81	13	Yes	Yes
MORDORIMort (2014)	Malawi	59.6 (67.2)	40.3 (44.4)	65.4	18.1	44.5	97	97	91	91	87	No	Yes*
MORDORIMort (2014)	Tanzania	56.7 (62.5)	40.1 (43.4)	49.4	8.7	29.7	99	99	97	97	93	No	Yes
MORDORII MortY5 (2017)	Niger	**124.3 (141.7)**	**62.7 (68.8)**	46.6	23.5	16.5	92	95	85	85	85	Yes	Yes
CHAT (2019)	Burkina Faso	**87.3 (119.5)**	53.8 **(67.3)**	49.0	60.1	50.7	98	95	91	91	91	Yes	Yes
LAKANA (2020)	Mali	**99.7 (122.1)**	**62.9 (73.1)**	80.7	30.1	56.0	78	75	70	70	66	Yes	Yes*
AVENIR (2020)	Niger	**121.0 (144.1)**	**61.6 (69.6)**	47.7	24.0	17.9	94	93	81	81	81	Yes	Yes
MIRAMA (2021)	Burkina Faso	**81.4 (119.4)**	51.2 **(67.3)**	49.0	63.6	54.1	98	95	91	91	66	Yes	Yes
AVENIR (Programmatic Trial, 2022)	Niger	**117.3 (147.7)**	**60.3 (70.8)**	48.9	24.1	18.9	95	96	84	84	84	Yes	Yes

BCG = Bacille Calmette-Guérin vaccine, DTP-1 = First dose of diphtheria-tetanus-pertussis vaccine, DTP-3 = Third dose of diphtheria-tetanus-pertussis vaccine, Hib = *Haemophilus influenzae* type B, PCV = Pneumococcal conjugate vaccine, SMC =

Seasonal malaria chemoprevention, U5 = Under-five, NA = Not available for the given year. **Bolded** cells indicate mortality rates which are above the thresholds recommended by the WHO for consideration of MDA-azithromycin to reduce childhood mortality (> 60 per 1000 live births for infant mortality and > 80 per 1000 live births for under-five mortality).

*The WHO validated trachoma elimination in Malawi in 2022 and in Mali in 2023 [1].

Data sources: UN Inter-agency Group for Child Mortality Estimation, 2023 (mortality estimates); WHO/UNICEF Joint Monitoring Programme, 2023 (WASH indicators); WHO/UNICEF estimates of national immunization coverage, 2023 revision (immunization indicators); World Malaria Report, 2023 (SMC use); WHO Global Health Observatory (trachoma status)

[1] WHO congratulates Benin and Mali for eliminating trachoma as a public health problem 2023. https://www.who.int/news/item/16-05-2023-who-congratulates-benin-and-mali-for-eliminating-trachoma-as-a-public-health-problem (accessed August 20, 2023).
